# Rational design of inhaled antibacterials targeting alveolar macrophages for efficient killing of resistant bacteria associated with lower respiratory tract infections (LRTIs)

**DOI:** 10.1080/10717544.2026.2752706

**Published:** 2026-11-03

**Authors:** Nazrul Islam, Emad L. Izake, Kirsten Spann, Yin Xiao, Timothy J. Wells

**Affiliations:** a Pharmacy Discipline, School of Clinical Sciences, Faculty of Health, Queensland University of Technology, Brisbane, QLD, Australia; b Centre for Immunology and Infection Control (CIIC), Faculty of Health, Queensland University of Technology, Brisbane, QLD, Australia; c School of Chemistry and Physics, Faculty of Science, Queensland University of Technology, Brisbane, QLD, Australia; d School of Medicine and Dentistry & Institute for Biomedicine and Glycomics, Griffith University, Gold Coast Campus, QLD, Australia; e Frazer Institute, Faculty of Medicine, The University of Queensland, Brisbane, QLD, Australia

**Keywords:** Alveolar macrophages (AM), lower respiratory tract infections (LRTIs), inhaled antibiotics, antibiotic resistant, intracellular bacteria, AM receptors, targeted delivery

## Abstract

Lower respiratory tract infections (LRTIs), caused by antibiotic-resistant bacteria, are a leading cause of global death and morbidity. One of the principal limitations in treating respiratory bacterial infections is inefficient delivery and appropriate therapeutic drug concentrations to infected cells, as required to kill resistant bacteria. Inhaled antibiotic delivery technology specifically delivers antibacterials directly into the infected cells of the lungs and provides the required therapeutic concentration at a very low delivered dose, with a greatly reduced risk of systemic adverse effects. An alveolar macrophage (AM)-targeted antibiotic delivery strategy utilizing receptor-ligand interactions or stimuli-responsive mechanisms of drug release demonstrated efficient killing of intracellular bacteria. The rational design of antibacterials or formulations for inhalation is the key to achieving this goal. Based on ligand chemistry, decorating antibiotic carriers with ligands that target specific AM receptors is a novel strategy to ensure precise antibiotic delivery to the site of infection while reducing both the side effects associated with high systemic doses and the risk of further promoting antibiotic resistance. This review highlights advances in AM-targeted inhaled antibacterials, identification of appropriate ligands for specific AM receptors, mechanisms of interaction, and subsequent release of antibiotics through carrier degradation, pH-responsive behavior, or enzymatic cleavage to eradicate resistant bacteria. Finally, we discuss the ongoing challenges and future directions for AM-targeted inhaled antibacterials against LRTIs.

## 1. Introduction

Effective treatment of lung infections, especially lower respiratory tract infections (LRTIs) associated with chronic obstructive pulmonary disease (COPD), cystic fibrosis (CF), and non-CF bronchiectasis (NCFB), poses a significant challenge because of the development of antibiotic resistance following long-term inefficient use of currently available antibiotic delivery strategies (Feng et al. 2023; Islam and Reid 2024). *Staphylococcus aureus, Mycobacterium sp., Burkholderia sp.,* and *Pseudomonas aeruginosa* are the most commonly identified pathogens that are difficult to treat using currently available antibiotic dosage forms. Pulmonary, rather than oral or systemic, drug delivery technology is a compelling strategy to directly deliver antibiotics to infected cells within the lungs. This enables efficient killing of bacteria at a lower dose than systemic delivery, which has systemic adverse effects. Although macrophages are the primary lung cells infected by bacteria due to their phagocytic activity, airway epithelial cells can also serve as important reservoirs for several clinically relevant pathogens, including *P. aeruginosa* and *S. aureus,* highlighting the need for therapeutic strategies that target both cellular reservoirs.

Alveolar macrophages (AM) reside in alveolar sacs and serve as the first line of defense against inhaled microorganisms, airborne particulate matter, and environmental toxins. Macrophage-targeted antibiotic delivery strategies show promise as they allow site-specific release of antibiotics for efficient killing of intracellular bacteria, while reducing drug toxicity and deleterious side effects (Liu et al. 2023; Chae et al. 2023). For example, Zlotnikov et al. (2022) successfully studied antibiotic-loaded mannose-decorated carriers targeting the CD206 macrophage mannose receptor, and the developed system effectively recognized these receptors with enhanced cellular uptake and antibacterial action of moxifloxacin. The authors synthesized a series of mannosylated polymer-based conjugate carriers using a natural polysaccharide (mannan), polyethyleneimine (PEI), and cyclodextrin, which are suitable for intracellular antibiotic delivery. These findings highlight that mannosylation is a viable strategy for targeted drug delivery to overcome macrophage-associated infections. Previously, moxifloxacin-loaded ROS-responsive 4-(hydroxymethyl) phenylboronic acid pinacol ester-modified *α*-cyclodextrin (Oxi-αCD) NPs for active targeting of folate receptors on AMs were demonstrated (Wang et al. 2019). The developed folic acid (FA)-modified NPs (Max/FA-Oxi-αCD) for internalizing AMs through overexpressed folate receptors (FRs) showed effective killing of intracellular *P. aeruginosa* in a mouse model. The NPs penetrated the mucous barrier and produced a controlled release of antibiotics for up to 25 hours. The interactions of decorated nanoparticles with the mucus barrier and mucociliary clearance mechanisms in the lungs of patients with COPD and CF have not yet been fully elucidated. Consequently, further investigations are warranted to overcome these limitations and evaluate their clinical applicability. AM-targeted mannose-decorated solid-lipid nanoparticles of rifampicin (Bera et al. 2024) and ciprofloxacin-loaded polymeric prodrug (Chavas et al. 2021) produced improved AM uptake of drugs. In a mouse model, the inhaled ciprofloxacin prodrug produced significant effects against clinical isolates of *Burkholderia pseudomallei* (Chavas et al. 2021).

The inhaled targeted delivery strategy for antibiotics improves intracellular drug delivery (Chae et al. 2023; Islam and Reid 2024) and enhances antibacterial effectiveness against resistant pathogens (Islam and Collet 2026) residing inside macrophages. Although the referenced studies provide valuable insights into pulmonary antibiotic delivery for respiratory tract infections, our manuscript uniquely highlights the engineering of receptor-targeted engineered nanoparticles for alveolar macrophages, resulting in site-specific drug release and increased intracellular bacterial eradication. The potential future applications of these targeted strategies are also discussed.

## 2. Lower respiratory tract infections (LRTIs)

Lower respiratory tract infections (LRTIs) are one of the leading causes of global morbidity and mortality owing to their increased antibiotic resistance (Ding et al. 2021; Zhu et al. 2023). In addition to acute infections, LRTIs exacerbate chronic lung conditions. The main causative bacteria in these chronic conditions are *S. aureus* and *P. aeruginosa*. These organisms can form biofilms within the lungs, which significantly limits the efficacy of antibiotics and renders treatment challenging. Other common bacteria associated with chronic lung diseases are non-tuberculosis *Mycobacteria spp,* and *Burkholderia spp* which are naturally multidrug-resistant and notoriously difficult to eradicate with current antibiotic treatments, primarily as they can cause intracellular infections (Cohen et al. 2018). Both genera of bacteria can enter and replicate in AMs, allowing both survival and dissemination of bacteria (Martin and Mohr 2000). AMs form the first line of defense against these pathogens and are responsible for their phagocytic uptake and bactericidal effects. However, increasing evidence highlights that many extracellular pathogens can survive intracellularly in AMs and thus evade antimicrobial immune responses. Both *S. aureus* and *P. aeruginosa* can survive within macrophages, which promotes bacterial growth and persistence (Pidwill et al. 2020). Biofilm formation and bacterial persistence within AMs make the eradication of these pathogens difficult using standard systemic antibiotic delivery, which cannot penetrate these cells. Additionally, these bacteria are currently resistant to most antibiotics through rapid mutational adaptation in response to the inappropriate overuse of antibiotics (Darby et al. 2023). There is an unmet need for effective strategies for managing LRTIs, including targeted drug delivery strategies that allow accurate targeting of pathogens with enhanced killing at a low dose.

## 3. Current treatment systems and their limitations

Currently, treatment for intracellular bacteria involves high doses of antibiotics administered intravenously or orally. These high doses are associated with off-target adverse effects, as most antibiotics are unable to reach the bacteria within infected AMs. Subinhibitory concentrations of the delivered antibiotics from the dosage forms affect the biochemical and biophysical properties of developing biofilms of bacteria, and the effectiveness of antibiotics is seriously affected (Chen et al. 2021). Overuse of intravenous and oral antibiotics has led to the development of antibiotic resistance and makes treatment of bacterial infections difficult (Lechtzin et al. 2006). Organisms such as *P. aeruginosa* and *S. aureus* can form biofilms that require high doses of antibiotics to penetrate a sufficient dose to inhibit bacterial growth, which is difficult to achieve using systemic therapies. Inhaled antibiotic systems (Cipolla and Chan 2013) allow for more targeted delivery of antibacterials to the lung, specifically to the AM harboring intracellular bacteria (Lim et al. 2021). Recently, strategies to develop inhaled nanoparticulate antibiotic formulations to directly deliver drugs to infected lung macrophages and kill antibiotic-resistant bacteria have been reported with future potential (Lim et al. 2021; Islam and Reid 2024; Suraweera et al. 2025). A brief description of the pulmonary drug delivery technology is presented in the next section (Section 4).

## 4. Pulmonary drug delivery technology

Pulmonary drug delivery technology ensures targeted delivery of drugs into the lungs to the site of infection, producing the required therapeutic action at a significantly low dose. Direct delivery of drugs into the lungs enables the use of lower doses with an equivalent therapeutic action compared to normal systemic administration due to the large surface area (~140 m^2^) and extremely thin (0.1–0.2 μm) absorptive pulmonary epithelium with good blood supply (Patton and Byron 2007). Lung delivery technology avoids fast-pass metabolism, which is a common problem for orally administered drugs. Furthermore, lung drug delivery ensures rapid onset of action and fewer systemic side​effects compared with oral or intravenous drug administration.

Aerosol deposition in the respiratory tract is governed primarily by the particle's aerodynamic diameter, which determines the relative contributions of inertial impaction, gravitational sedimentation, and Brownian diffusion (Löndahl et al. 2014; Morawska and Buonanno 2021). For deep lung deposition, particles with an aerodynamic diameter of approximately 1–5 μm are generally considered optimal because they can bypass the upper airways and deposit efficiently in the alveolar region by sedimentation. Particle engineering strategies therefore aim to tailor particle size, density, morphology, and surface properties to achieve a suitable aerodynamic behavior; for example, porous or low-density microparticles can possess a large geometric diameter while maintaining an aerodynamic diameter within the respirable range. In contrast, nanoparticles (<100–500 nm) exhibit high diffusivity and may penetrate deeply into the lung, but their deposition efficiency can be lower than that of optimally sized microparticles because many nanoparticles are exhaled before deposition. As a result, inhaled nanomedicines are often formulated into nanocomposite or nano-in-microparticle systems that combine the favorable aerosol performance of microparticles with the intracellular delivery advantages of nanoparticles (Takeuchi et al. 2020). Experimental and modeling studies consistently show that respirable microparticles (1–5 μm aerodynamic diameter) achieve the most efficient alveolar deposition, whereas nanoparticle deposition is dominated by diffusion and is highly dependent on particle size and breathing conditions.

### 4.1. Delivery devices

The successful delivery of inhaled antibiotics depends on the interplay between drug formulation, delivery devices, and patient-related factors (Zhou et al. 2015). The therapeutic efficacy of inhaled antibacterial agents is determined not only by the physicochemical properties of the formulation but also by the ability of the delivery device to efficiently deposit the intended dose within the lungs. Nebulizers, pressurized metered-dose inhalers (pMDIs), and dry powder inhalers (DPIs) are the three principal platforms currently used for pulmonary drug delivery (Islam and Gladki 2008). Although various nebulizer technologies are available, their suitability for delivering antibiotic formulations with diverse physicochemical characteristics remains incompletely understood. Consequently, dose adjustments and device-specific optimization may be necessary to achieve consistent pulmonary deposition and optimal antibacterial activity. Additionally, the stability of the antibiotic formulations in liquid form must be investigated.

Dry powder inhalers (DPIs) offer several advantages because they deliver antibiotics in a dry powder form and are generally portable and convenient for patients. However, efficient aerosolization and complete dispersion of powder from the capsule remain significant challenges, especially for high-dose formulations and combination antibiotic therapies. The development of high-dose, carrier-free DPI formulations requires advances in particle engineering, formulation design, and inhaler technology. Furthermore, DPI performance is highly dependent on the patient's inspiratory effort (Azouz and Chrystyn 2012), which can vary considerably among individuals and across disease states such as asthma, chronic obstructive pulmonary disease (COPD), and cystic fibrosis (CF). Despite ongoing progress in inhaler design, substantial knowledge gaps remain regarding the formulation of multiple antibacterial agents within a single inhalable product and their aerosolization performance when delivered using a device suitable for diverse patient populations, including children and older adults. Therefore, continued innovation and optimization of inhalation devices, including low-, medium-, and high-resistance DPIs, will be critical to maximizing pulmonary drug deposition, therapeutic efficacy, patient adherence, and the overall clinical success of inhaled antibacterial therapies.

As alveolar macrophages (AMs) play a key role in respiratory infections, this manuscript is dedicated to demonstrating macrophage-targeted inhalable antibiotic particle design, their preferential interactions with AMs, and the precise release of antibiotics for the efficient killing of intracellular resistant bacteria (Figure 1). There are several comprehensive review articles covering the strategies for pulmonary antibiotic delivery (Chae et al. 2023; Islam and Reid 2024); however, in this review, we will highlight the rational design of antibacterials for precision delivery into the lungs targeting AMs for intracellular bactericidal effects.

**Figure 1.**
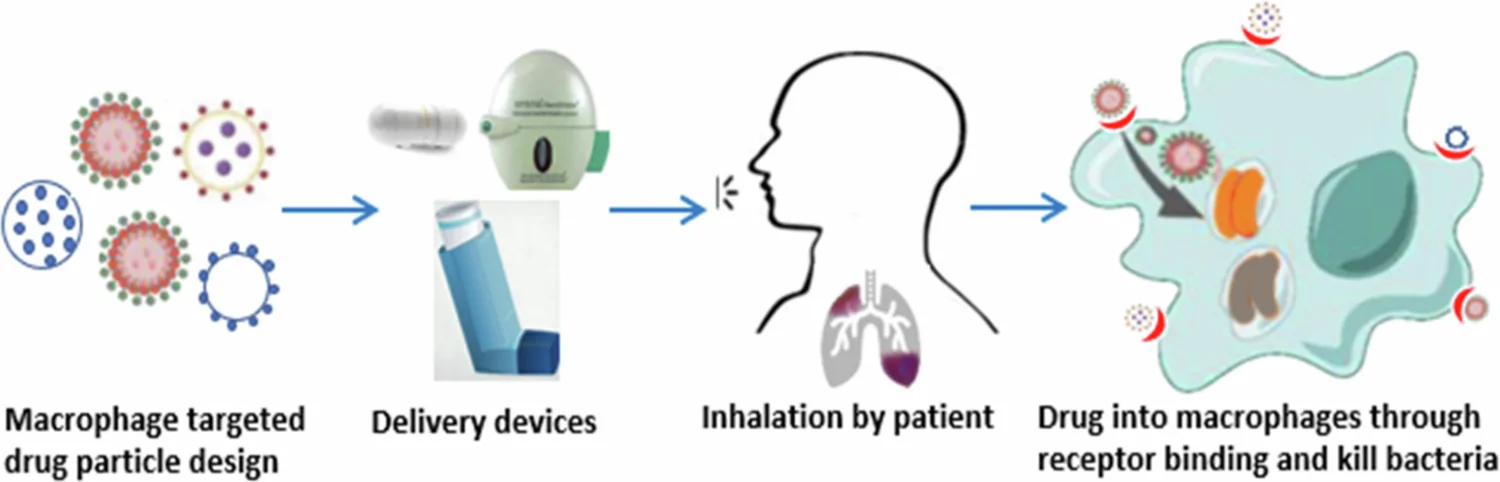
Schematic diagram of macrophage-targeted drug particle design, deposition into deep lungs by inhalation, and kill intracellular bacteria.

## 5. Why are alveolar macrophages (AMs) a novel target for antibiotic delivery?

Alveolar macrophages (AMs) are highly influenced by the local pulmonary environment and are responsible for the clearance of inhaled particles, microbes, and airway debris. In terms of microbial infection, pathogen identification occurs via pattern recognition receptors (PRRs) or toll-like receptors (TLRs), which activate macrophages to release M1-like proinflammatory mediators such as TNF-*α*, IL-1β, IL-6, IL-12, and kill intracellular bacteria (Martin and Frevert 2005; Melo et al. 2021). The reasons for targeting AMs for drug delivery are as follows.

*Specific receptors on AMs surfaces for targeted delivery*: There are various types of receptors available on the AMs surface, which are overexpressed during infection and recognize the invasion of pathogens in infected cells (Hirayama et al. 2018). AM-targeted antibiotic delivery systems are based on decorated drug nanoparticles with specific ligands that selectively bind to receptors on the cell surface, release antibiotics in AMs, and efficiently kill intracellular bacteria.

*AMs are easy to access:* AMs are easy to access via the oropharyngeal route and their intrinsic function of taking up foreign particles/microorganisms can be leveraged by inhaled drug delivery technology. AMs serve as the first line of defense against inhaled microorganisms, airborne particulate matter, and environmental toxins. They are activated by infectious and injurious agents to release large quantities of cytokines, which are responsible for clearing pathogens. Therefore, AMs are considered attractive therapeutic targets for the efficient killing of intracellular resistant bacteria.

*AMs are available at the airway-tissue interface*: AMs are available at the airway-tissue interface and sit at a unique intersection between the airway and parenchymal tissue from where gaseous exchange occurs. This intimate positioning between the airways and vasculature allows AMs to integrate signals originating from both locations due to injury or infection, and is easy to target with inhaled drugs.

AM-targeted inhaled antibiotics are currently best considered as an adjunct to systemic therapy rather than a replacement (Flume and VanDevanter 2015). While targeted pulmonary delivery can substantially increase antibiotic exposure at the primary site of infection and may permit future dose optimization, there is insufficient evidence that systemic antibiotic doses can be safely reduced without compromising overall therapeutic efficacy. Further pharmacokinetic-pharmacodynamic and clinical studies are required to define whether targeted inhaled formulations can support systemic dose reduction while maintaining bacterial eradication and preventing resistance development.

## 6. Design of AMs targeted antibiotic delivery strategies

AM-targeted antibiotic delivery strategies ensure uniform distribution of the delivered antibiotics in the intracellular compartment and efficiently kill intracellular bacteria. After macrophage internalization, targeted antibiotic formulations are generally trafficked to endosomal and lysosomal compartments (Wong et al. 2017). Depending on the carrier design, lysosomal processing may either degrade the formulation or facilitate controlled drug release. The therapeutic objective is to achieve effective antibiotic release within phagolysosomes compartments harboring intracellular bacteria rather than loss through lysosomal degradation. Although this concept is widely accepted, direct comparative evidence demonstrating efficient intraphagosomally drug release for macrophage-targeted inhaled antibiotics remains limited. Therefore, the rational design of antibacterials with appropriate formulations and the ability of the designed antibiotic delivery system to target subcellular locations would be the most important goals.

### 6.1. Ligand conjugation to target a receptor on AMs

Various techniques have been used to design antibacterial or micro/nanoparticle formulations (Lee et al. 2015; Chae et al. 2023) of antibiotics that interact with specific receptors on AMs and trigger drug release within subcellular regions. Designing a drug with carriers coupled with specific ligands that interact with the selective receptors on the AMs surface and release encapsulated/loaded drugs into the cells is one of them. Appropriate attachment of specific ligands (targeting moiety) to the carrier surface directs them to recognize and bind to the target cells. Examples of AMs receptors, the chemistries of specific ligands, and drug release/uptake mechanisms are summarized in Table 1.

**Table 1.** Ligand–receptor systems on alveolar macrophages with suitable linker chemistries and their typical release triggers.

Ligand chemistry	Primary AM receptor	Uptake mechanism	References
Mannose, mannosylated polymers/clusters	CD206 (C-type lectin)	Receptor-mediated endocytosis	Zlotnikov and Kudryashova (2022)
Fucose, fucosylated ligands	DC-SIGN (CD209) C-type lectin	Endocytosis via carbohydrate recognition	van der Zande et al. (2021); Zlotnikov and Kudryashova (2022)
*β*-Glucans, yeast-derived polysaccharides	Dectin-1 (Pattern-recognition receptor)	Phagocytic uptake; strong activation of AMs	Scur et al. (2022)
Opsonins (IgG fragments, Fc-bearing ligands)	Fcγ Receptors (FcγRI, II, III) (Immunoglobulin receptors)	Fc-mediated phagocytosis	Hou et al. (2025)
Lipophilic ligands (modified lipids, mycolic acid mimics)	CD36, SR-B1 (Lipid scavenger receptors)	Lipid-mediated uptake	Scur et al. (2022)
Cationic polymers (chitosan, PEI derivatives)	Non-specific electrostatic uptake/Charge-mediated	Macropinocytosis, phagocytosis	Zlotnikov et al. (2022)
Complement-binding ligands (e.g. C3b-decorated particles)	Complement Receptor for C3b	Opsonin-mediated uptake.	Dixit et al. (1982)

Targeting the AMs mannose receptor (CD206) is an effective strategy for antibiotic delivery for efficient killing of intracellular bacteria, as CD206 is expressed at high levels in infected macrophage cells (Chen et al. 2019; Zlotnikov and Kudryashova 2022; Chae et al. 2023). For example, mannose-decorated, rifampicin-loaded solid lipid nanoparticles (Man-RIF-SLNPs) have been shown to effectively bind mannose receptors on infected alveolar macrophages, enabling targeted antibiotic release and efficient killing of intracellular *Mycobacterium intracellulare* (Figure 2) (Chen et al. 2019; Zlotnikov and Kudryashova 2022; Chae et al. 2023). The authors reported that Man-RIF-SLNPs controlled rifampicin release and extended formulation stability. In addition, these nanoparticles exhibited markedly stronger antibiofilm and antibacterial activities than free rifampicin at 100 μg/mL. Cytotoxicity studies revealed that Man-RIF-SLNPs maintained over 85% viability in M2 macrophages at the same concentration, indicating that mannose functionalization did not induce significant toxicity in the immune cells.

**Figure 2.**
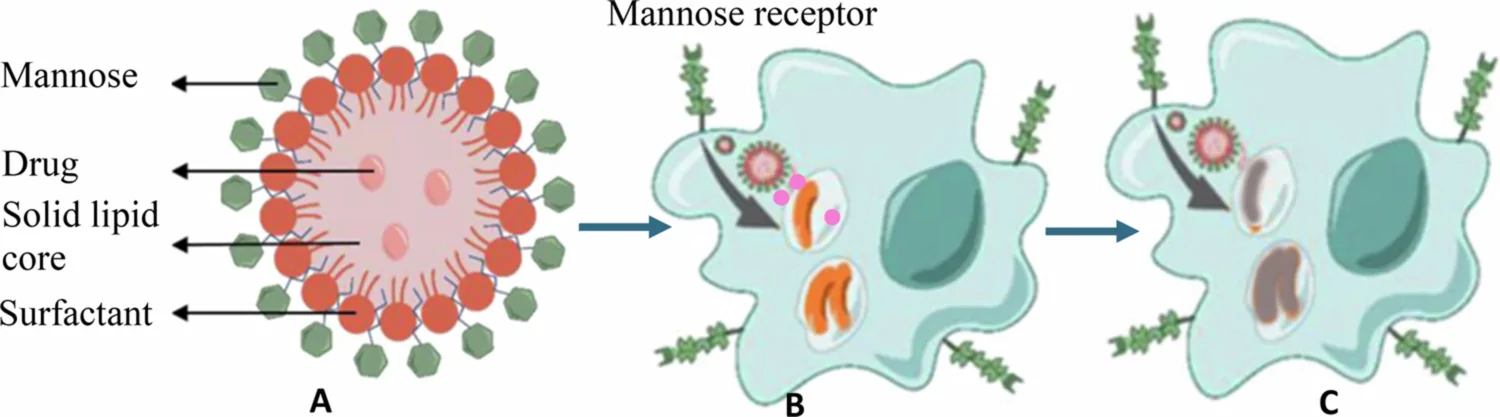
Targeted antibiotic release into the infected macrophage following interacting with the mannose receptor. A. Antibiotic-loaded mannose-decorated solid lipid NPs; B. Interaction of mannosylated carrier with the mannose receptor on the macrophage and drug release around the live bacteria, C. Dead bacteria, and survival of macrophage. Reproduced from Ref (Chae et al. 2023).

Dectin-1 receptor-targeted antibiotic delivery is an emerging strategy for improving the treatment of lung infections by selectively directing antimicrobial agents to infected lung cells (Scur et al. 2022). By functionalizing nanoparticles, liposomes, or polymeric drug carriers with *β*-glucan ligands or Dectin-1binding moieties, antibiotics can be preferentially delivered to Dectin-1expressing cells within the infected lung. This targeted approach enhances local drug accumulation at the site of infection, promotes uptake by phagocytic immune cells, and may improve intracellular clearance of pathogens while reducing systemic drug exposure and associated toxicity. For lung infections, Dectin-1 targeting is scientifically attractive because it combines drug delivery with immune modulation and directly targets phagocytic cells involved in pathogen clearance; however, current literature largely consists of animal and in vitro studies, and there are no widely reported clinical trials for lung infections.

Fc receptor-targeted antibiotic delivery is the use of IgG or Fc-functionalized antibiotic carriers designed to exploit Fcγ receptors expressed on macrophages, thereby enhancing intracellular antibiotic delivery to infected phagocytic cells (Hou et al. 2025). From a critical perspective, Fc receptor-targeted delivery offers several advantages, including improved cellular specificity, increased intracellular antibiotic concentrations, and the potential to reduce systemic toxicity. However, its clinical translation remains challenging because Fc receptor expression varies with macrophage phenotype, disease state, and inflammatory conditions, which can lead to inconsistent targeting efficiency.

Among currently investigated receptor-targeting strategies for inhaled antibiotic delivery, mannose receptor (CD206) targeting possesses the strongest evidence base for improving intracellular pharmacokinetics, largely because multivalent mannose ligands efficiently promote macrophage uptake and sustained intracellular drug retention (Paurevic et al. 2024). Dectin-1 targeting offers the advantage of strong phagocytic signaling and intracellular delivery but lacks extensive PK/PD validation in inhaled antibiotic systems (Mata-Martinez et al. 2022). Folate receptor-*β* targeting provides highly specific recognition of activated macrophages and may enhance intracellular localization (Steinz et al. 2022), although direct evidence demonstrating prolonged pulmonary intracellular antibiotic exposure remains relatively limited. Overall, successful receptor-targeted antibiotic delivery depends not only on receptor selection but also on ligand valency, linker design, spatial presentation, intracellular trafficking, and controlled drug release within bacteria-containing compartments.

In comparison, Dectin-1-targeted carriers can enhance uptake by phagocytic cells through receptor-mediated endocytosis, thereby improving intracellular drug delivery to pathogens that reside within host cells. Mannose receptor (CD206)-targeted systems similarly facilitate macrophage uptake but primarily target macrophage populations expressing CD206, which may vary depending on the disease state and inflammatory microenvironment. Fcγ receptor-targeted approaches can achieve efficient internalization by immune cells; however, their performance may be influenced by receptor heterogeneity and the potential for receptor-mediated immune activation. Consequently, each targeting strategy presents distinct advantages and limitations that should be considered according to the intended therapeutic application.

Dai et al. (2024) developed vancomycin (Van)-encapsulated nanoparticles targeting bacteria-infected macrophages to eliminate intracellular bacterial infections via antibiotic release, upregulation of intracellular reactive oxygen species (ROS) levels, and proinflammatory responses to enhance antibacterial activity. Vancomycin-loaded NPs (ox-Dex-Se/Van), formed through the reaction of the amino group on selenocystamine dihydrochloride and the aldehyde group on oxidized dextran to ensure macrophage uptake via endocytosis mediated by the mannose receptor. The ox-Dex-Se/Van NPs along with 1 mM H_2_O_2_, a potent growth inhibitor of *P. aeruginosa* at a concentration of 17.2 ug/mL following increased ROS generation influenced by H_2_O_2_. NPs without H_2_O_2_ did not show significant antibacterial activity at the same concentration. These activities of ox-Dex-Se/Van NPs contributed to their higher *P. aeruginosa* killing activity compared to that of free vancomycin or ox-Dex-Se/Van without H_2_O_2_.

Macrophage cell membrane-based nanoparticulate drug delivery for targeted delivery of drugs for efficient treatment of diseases has been reported (Wu et al. 2022). Li et al. (2020) demonstrated that macrophage membrane-coated triclosan and ciprofloxacin NPs were internalized by specific toll-like receptors and successfully killed intracellular *S. aureus*. Efficient killing of intracellular *S. aureus* by macrophage membrane receptor-coated gold-silver nanocages has also been reported (Wang et al. 2018). Recently, using components from bacterial outer membrane vesicles, Cai et al. (2025) developed M1-targeting drug delivery system to enhance target specificity by decorating gold nanocage (AuNC) NPs with ligands, which demonstrated high affinity for macrophage surface receptors in pulmonary environments. The hybridized NPs were selectively engulfed by macrophages through the phagocytosis-related receptors CD64 and CD14, which are highly expressed in the infected macrophages. Although this work showed a high-level selective M1-targeted drug delivery technology, no antibacterial studies have been conducted to ensure preferential killing of intracellular bacteria. These technologies are innovatiove for receptor-specific macrophage-targeted drug delivery, which may be applicable to efficiently kill intracellular resistant bacteria; however, extensive studies on the toxicity and safety of these NPs are warranted for pulmonary delivery of decorated antibiotics.

Targeted delivery of ciprofloxacin by AM has been demonstrated with effective intracellular antibacterial outcomes against clinical isolates of *Burkholderia pseudomallei* (Chavas et al. 2021). The authors developed a ciprofloxacin prodrug consisting of multivalent mannose ligands that target mannose receptors and a protease-cleavable dipeptide motif that links ciprofloxacin to the polymer backbone. This motif is cleaved by intracellular proteases, allowing the release of antibiotics into AMs (Figure 3). As depicted in the figure, the drugamer is internalized in AMs via interaction with the mannose receptor (active targeting) following endocytosis (1). This allows the release of ciprofloxacin from the drugamer by breaking the protease-cleavable dipeptide linker triggered by the intracellular protease enzyme (2). The authors intratracheally administered the drugamer in mice via a microsprayer, collected the lung macrophages through bronchoalveolar lavage, and determined ciprofloxacin by HPLC. Ciprofloxacin was released from the drugamer into the AMs and maintained sustained action against organisms for over 5 days. This technique applied active targeting of AMs and a passive mechanism of antibiotic release in infected cells and eradicated intracellular *B. pseudomallei and B. thailandensis*. The designed ciprofloxacin drugamer offered full protection to the mice, and 80% of the drugamer-treated group completely eradicated the intracellular bacteria. This technology serves dual purposes (active and passive targeting AMs) with viable outcomes for AM-targeted antibiotic delivery and successful killing of intracellular bacteria.

**Figure 3.**
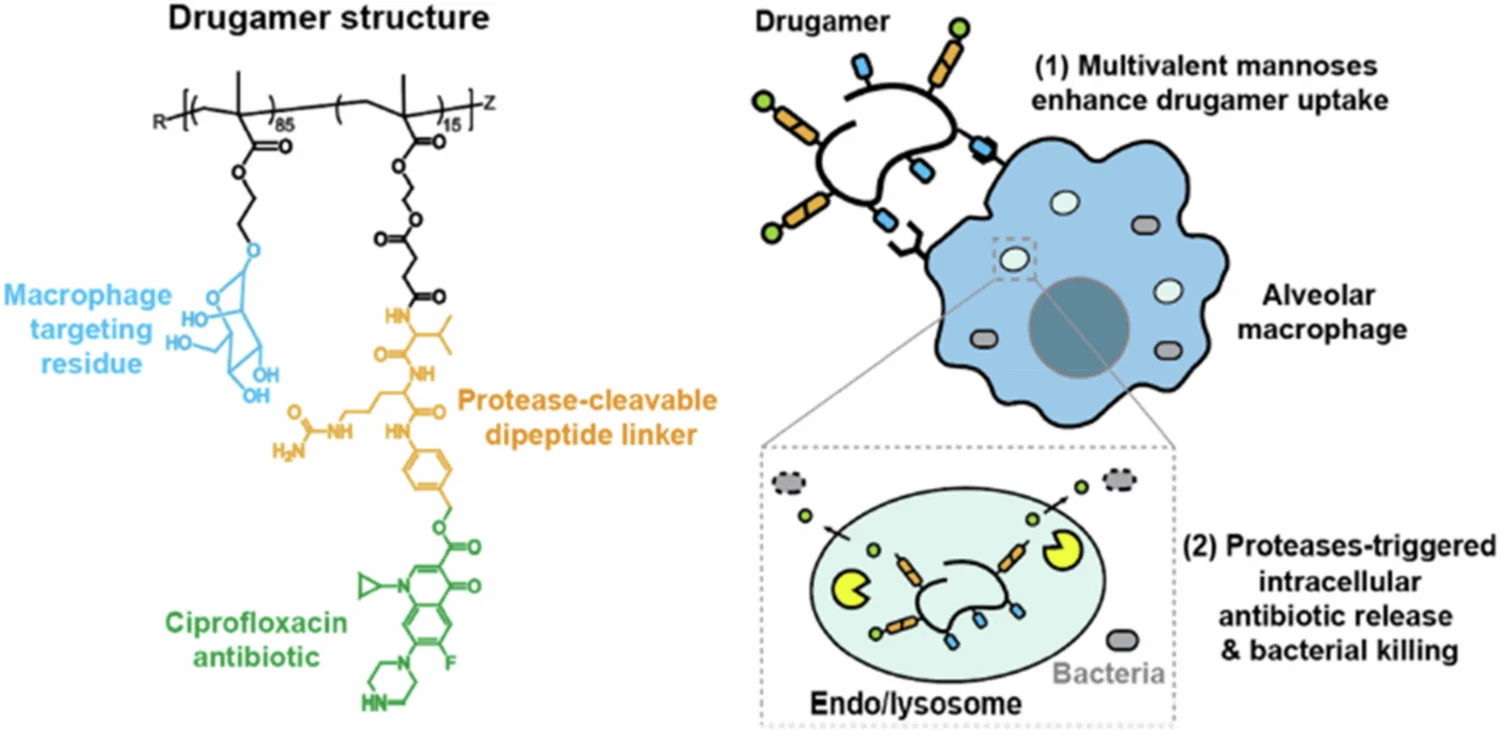
Macrophage-targeted delivery of ciprofloxacin from a complex known as drugamer. (1) drugamer internalization by alveolar macrophages following binding with mannose receptors; (2) ciprofloxacin released triggered by enzyme protease in the AM, allowing release of ciprofloxacin for killing intracellular bacteria. Adapted from ref (Chavas et al. 2021).

While quantitative information regarding intracellular antibiotic concentrations and sustained supra-MIC exposure is currently limited, the macrophage-targeted formulation exhibited significantly enhanced uptake by macrophages compared with the unconjugated formulation. Previous studies have demonstrated that macrophage-targeted antibiotic delivery systems can substantially increase intracellular drug exposure. For example, Su et al. (2018) reported that a mannose-targeted ciprofloxacin prodrug achieved more than a 10-fold increase in sustained intracellular drug levels within alveolar macrophages and maintained concentrations above the MIC for at least 48 hours compared with free ciprofloxacin, supporting the potential of macrophage-targeted strategies to improve treatment of intracellular pulmonary infections. This finding supports the concept that active receptor-mediated targeting may provide pharmacodynamic advantages over passive uptake alone by enhancing intracellular drug delivery to the site of infection. Future studies using infected primary macrophages are warranted to directly quantify intracellular drug levels and determine pharmacokinetic/pharmacodynamic parameters, including intracellular AUC, Cmax, and time above MIC, to further establish the therapeutic benefits of macrophage-targeted inhaled antibiotic delivery.

It should be noted that while receptor-binding affinity contributes to enhanced receptor-mediated macrophage uptake and intracellular drug accumulation, improved bioavailability and intracellular retention are governed by multiple factors, including intracellular trafficking, drug release kinetics, subcellular localization, and retention within bacteria-containing compartments. However, superior macrophage receptor affinity does not necessarily translate into enhanced drug bioavailability (Pea 2018). Nevertheless, receptor-targeted formulations have consistently demonstrated greater macrophage uptake and more sustained intracellular drug exposure than non-targeted systems (Su et al. 2018), suggesting a potential therapeutic advantage.

### 6.2. Design of antibacterials for non-receptor targeting of AMs

Drug delivery via passive targeting is based on non-receptor-mediated drug release into macrophages to eradicate intracellular bacteria. It is also known as stimulus-responsive drug delivery technology. The most common approach for passive targeting of drugs into AMs is to modulate the physicochemical properties (particle size, surface charge, solubility, and morphology) of drugs and the microenvironment (pH, enzyme, etc.) of AM; however, this technique lacks targeted drug release into specific cells. Yang et al. (2022) developed a dual pH-responsive polymer (poly[(3-phenylprop-2-ene-1,1-diyl)bis(oxy)bis(ethane-2,1-diyl)diacrylate-co-*N*-aminoethylpiperazine] (PCA)) NPs, to eradicate intracellular bacteria by activating M1 polarization, leading to the destruction of the bacterial cell walls (Figure 4). The authors demonstrated that the presence of acid-labile acetal linkages and tertiary amine groups in the polymer backbone facilitated biodegradable PCA dual pH response activity that produced an acid-induced increase in positive charge and released the drug in an acidic environment (pH 5). The NPs passively up taken by AMs (endocytosis) and released cinnamaldehyde, which generated ROS in the AMs and upregulated M1 polarization leading to the successful killing of intracellular *S. aureus* by disrupting bacterial cell membrane integrity at a concentration of 31.3 μg/mL and complete eradication of bacteria was obtained at 62.5 μg/mL.

**Figure 4.**
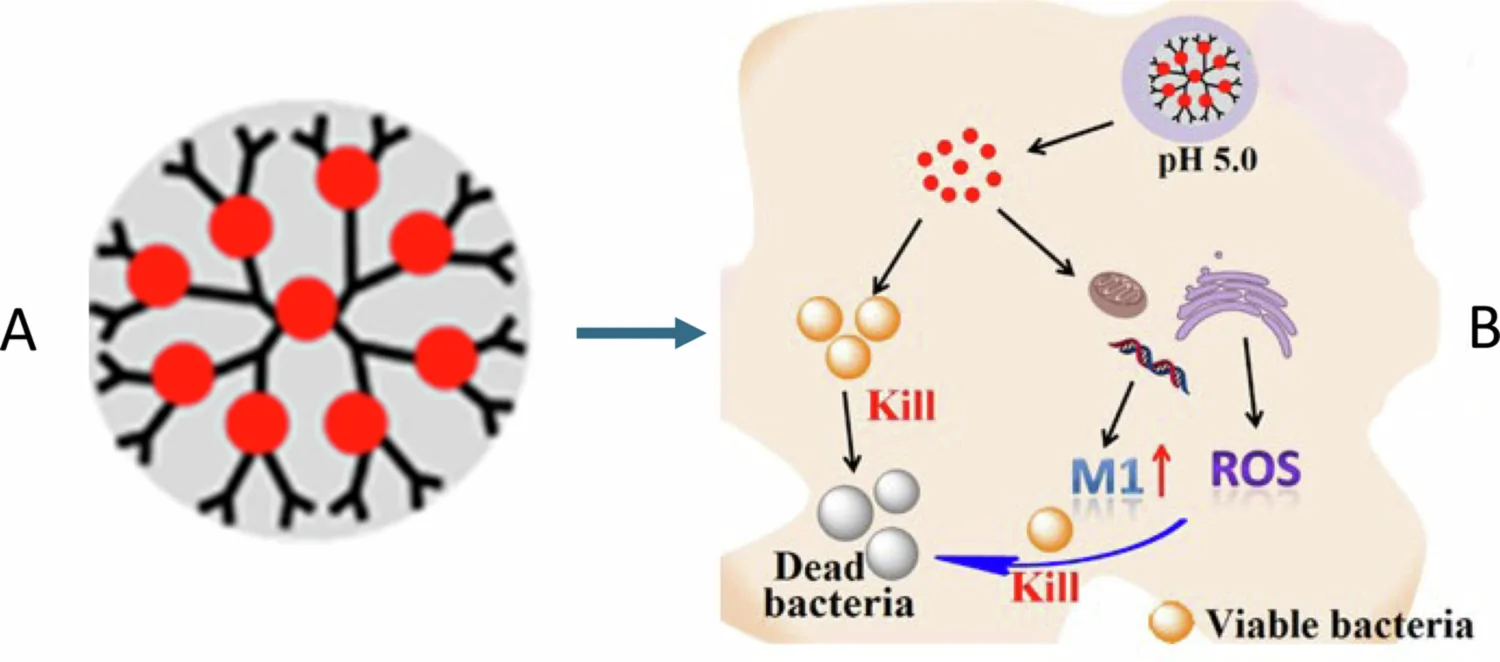
pH-responsive hyperbranched polymer NPs (A), drug released at acidic environment, and eradicate intracellular bacteria by activating M1 polarization and enhancing ROS activities (B). Reproduced from Ref. (Yang et al. 2022).

Similar effects of increased M1 polarization by ciprofloxacin-loaded mesoporous nanozymes for successful killing of *P. aeruginosa* have been observed (Zhu et al. 2023). The nanozymes were prepared by the physical adsorption of ciprofloxacin (CIP) or glucose oxides (GOx) and MRu2 NPs and modified with hyaluronic acid (HA). The authors suggested that the prepared nanoenzyme (HA@MRuO_2_-Cip/Gox) disrupted the bacterial biofilm, entered the bacterial cells, cleaved extracellular DNA, generated a large amount of ROS, and efficiently killed bacteria. The antibacterial efficiency of HA@MRuO_2_-Cip/Gox against *P. aeruginosa*. HA@MRuO_2_-Cip/Gox enhanced macrophage internalization of bacteria and induced M1 polarization, leading to antibacterial activity. Additionally, the cell viability of HA@MRuO_2_-Cip/Gox was approximately 90% at high concentrations up to 500 μg/mL. Moreover, after injecting a high dose (250 μg/mL) of HA@MRuO_2_-Cip/Gox into healthy BALB/c mice, no significant changes were found in the heart, liver, spleen, lung, and kidney, indicating a high level of safety. Furthermore, the in vivo study confirmed that HA@MRuO_2_-Cip/Gox can trigger macrophage-related immunity and effectively alleviate lung infections.

While targeting AM to induce M1 polarization may enhance intracellular bacterial clearance, excessive or sustained M1 activation can amplify the release of pro-inflammatory mediators such as TNF-*α*, IL-1β, and IL-6, promoting neutrophil recruitment, tissue damage, and alveolar-capillary barrier dysfunction, thereby increasing the risk of exacerbating inflammatory lung injury (Wang et al. 2023). Therefore, careful optimization of inhalable particle design is necessary to maximize antibacterial efficacy while minimizing excessive M1-mediated inflammatory responses; otherwise, adjunctive anti-inflammatory therapy may be required to prevent exacerbation of lung injury.

Overall, active targeting has the potential to enhance intracellular pharmacokinetics beyond passive uptake by promoting receptor-mediated internalization, increasing macrophage-associated drug accumulation, and prolonging intracellular retention (Andrade et al. 2021). However, direct quantitative comparisons between targeted and non-targeted inhaled antibiotic formulations remain scarce. Consequently, it is not yet clear to what extent active targeting improves intracellular antibiotic exposure beyond that achieved through passive uptake following conventional inhalation. Future studies should directly compare intracellular drug concentrations, key intracellular pharmacokinetic parameters, and the duration of exposure above the minimum inhibitory concentration (MIC) in infected macrophages treated with targeted and non-targeted formulations. Such investigations would provide definitive evidence of the added value of active macrophage targeting and help establish whether enhanced intracellular exposure translates into improved pharmacodynamic outcomes and therapeutic efficacy.

### 6.3. Design of particle surface charge modification

Modification of the surface charge plays a significant role in designing antibacterial agents for the efficient killing of intracellular bacteria. Cationic nanocarriers are designed with positively charged surface groups, such as amines and quaternary ammonium moieties, which strongly interact with the negatively charged biofilm matrix composed of extracellular DNA, proteins, and polysaccharides (Guo et al. 2023). Although cationic liposomal surfaces can enhance cellular uptake (Bilton et al. 2020; Wang 2021; Liu et al. 2023), this benefit often comes at the cost of increased macrophage clearance. Their electrostatic attraction to negatively charged AM improves drug penetration and intracellular delivery. In parallel, formulations incorporating enzymatically responsive carriers use linkers or surface coatings that are selectively cleaved by enzymes abundant within biofilms, such as bacterial proteases, lipases, or host-derived inflammatory enzymes (Zhu et al. 2023).

Liposomes, composed of a phospholipid bilayer and used to deliver both hydrophilic and hydrophobic drugs to target cells, are passively engulfed (endocytosis) by macrophages and sustained-release drugs (Zaru et al. 2009; Togami et al. 2013; Ferguson et al. 2023), thus prolonging the action of drugs in the infected areas, with efficient clearance of intracellular bacteria (Khatib et al. 2019; Bassetti et al. 2020). Depending on the particle size and surface charge of the liposome carrier (Liu et al. 2023), antibiotic formulations can penetrate the biofilm layer (Dong et al. 2015; Kluzek et al. 2022). Using a rat model, nebulized rifampicin-loaded liposomal NPs produced remarkable effects on *Mycobacterium avium* complex (MAC) residing in AM (Zaru et al. 2009). Similar effects of liposomal amikacin (Zhang et al. 2018) and ciprofloxacin (Blanchard et al. 2018) have been observed against non-tuberculous mycobacteria, *M. avium*. Although biocompatible liposomal-encapsulated antibiotics possess the properties required to interact with alveolar macrophages and release antibiotics within the macrophages to kill intracellular bacteria, it is imperative to clinically investigate the safety and efficacy of inhaled formulations for the effective treatment of LRTIs. A summary of macrophage-targeted antibiotic delivery systems is presented in Table 2.

Rifampicin is one of the most extensively investigated antibiotics for pulmonary delivery targeting AMs for lung infections. Although many studies have reported robust outcomes, research has largely focused on formulation development, aerosolization performance, drug deposition, and in vitro antibacterial activity. Moreover, the development of inhaled rifampicin remains predominantly at the preclinical stage, with limited evaluation in clinically relevant infection models and an absence of clinical studies assessing therapeutically relevant inhaled doses. Some other inhaled antibiotics formulation targeting different receptors (Table 2) showed interesting outcomes; however, their clinical relevance is not established yet. Despite encouraging progress, substantial translational and clinical evidence is required to establish the safety, efficacy, and clinical utility of inhaled antibacterial formulations.

A key limitation of murine intratracheal instillation models is that they do not accurately reproduce human pulmonary aerosol deposition, as the delivered dose is directly instilled into the airways rather than inhaled under physiological breathing conditions. Consequently, the distribution, regional lung deposition, and clearance of particles may differ substantially from those observed in humans. Murine alveolar macrophages differ from their human counterparts in receptor expression, activation states, cytokine production, and responses to pathogens and nanocarriers, which may affect the uptake and therapeutic performance of macrophage-targeted delivery systems. Therefore, although murine intratracheal instillation models provide valuable proof-of-concept data, their ability to predict human pulmonary deposition, pharmacokinetics, and clinical efficacy remains limited, highlighting the need for complementary inhalation studies in more clinically relevant animal models and, ultimately, clinical trials. To facilitate clinical translation, future studies should focus on safety evaluations, pharmacokinetic–pharmacodynamic characterization, and efficacy testing in clinically relevant pulmonary infection models. These investigations will provide the evidence necessary to advance inhaled antibiotic formulations into clinical trials, ultimately establishing their safety, therapeutic efficacy, and translational potential.

**Table 2.** AMs targeted antibiotic delivery systems with brief outcomes.

Antibiotic	Formulation	Outcomes	Ref.
Rifampicin	Mannosylated SLNPs targeting mannose receptors on AM	Antibacterial and antibiofilm activities against non-tubercular *M. intracellulare* inside macrophages	Chae et al. (2023)
Rifampicin	Mannosylated cationic lipid carriers for targeting mannose receptors	Achieved AMs-specific targeted delivery of rifampicin with high efficiency and no inflammatory response.	Song et al. (2015)
Vancomycin	Mannosyl ligands conjugated to PEG and polyphosphoester crosslinked	Preferentially entered MRSA-infected RAW264.7 macrophages and reduced the survival rate of intracellular bacteria	Xiong et al. (2012)
Rifampicin	Hyaluronic acid-tocopherol-micelles	Successful effects against *Mycobacterium tuberculosis* following AM uptake	Gao et al. (2015)
Rifampicin	Drug-loaded SLNPs with mucoadhesion property by chitosan	Improved AMs uptake of the drug with effective elimination of *M. tuberculosis*	Vieira et al. (2021)
Ciprofloxacin	Manosylated AMs targeted ciprofloxacin prodrug	Aerosolized formulation produced sustained release of drug in AMs and efficiently killed *Burkholderia pseudomallei* in mouse model	Chavas et al. (2021)
Bedaquiline	Drug encapsulated fucosylated liposomal NPs	The fucosylated antibiotic produced enhanced bacterial killing of nontuberculous mycobacterial infection	Huck et al. (2022)
Rifampicin and isoniazid	Mannose-decorated lipopolysaccharide	The nanoformulations produced safe and efficacious AMs targeted delivery of drugs against tuberculosis	Sumaila et al. (2023)
Rifampicin	Drug-loaded monnosylated SLNPs	The AMs targeted mannose-modified SLNPs preferentially up taken by macrophages; however, showed significant cytotoxicity on A549 cells	Bera et al. (2024)
Ofloxacin	Drug-loaded chitosan microsphere	Inhaled chitosan-based ofloxacin delivery increased antibacterial efficiency	Park et al. (2013)
Moxifloxacin	ROS-responsive drug-loaded ester-modified *α*-cyclodextrin coated with folic acid	Preferential accumulation of the drug in AMs through folate receptor with remarkable activity against intracellular *P. aeruginosa* in a mouse model	Wang et al. (2019)
Rifampicin	Rifampicin-loaded liposome NPs passively internalized into AM and released drug	Nebulized rifampicin-loaded liposomal nanoparticles produced remarkable effects on *M. avium* complex in rat lung AMs	Zaru et al. (2009)
Amikacin	Liposomal amikacin passively engulfed by AMs	The release of amikacin from inhaled liposomal suspension macrophages was much higher than that of intravenous free amikacin and successfully killed *M. avium* in a rat model	Zhang et al. (2018)

## 7. AMs targeted antibiotic delivery-challenges and future strategies

### 7.1. Challenges

Macrophages are well-suited targets for intracellular antibiotic delivery because of their biological characteristics and phagocytic properties; however, there are some challenges in the development of drug carriers targeting macrophages and releasing antibiotics in infected cells. Although various studies have demonstrated a potentially transformative strategy in macrophage-targeted drug delivery, several challenges must be overcome to achieve efficient therapeutic effects and clinical application with minimal toxic effects. The main challenges include the design of an engineered drug carrier system, antibiotic loading, selection of macrophage sources, and efficient delivery and release of drugs at the target site (Guo and Qian 2024). To eliminate intracellular bacteria, the designed drug carrier system must initially cross the cell membrane and release antibiotics into the bacterial cells, either through active targeted receptor interactions or stimuli-responsive targeted technology. Although the common strategies for specific cellular internalization of antibiotics by macrophages include receptor- and non-receptor-mediated, the most efficient way is to get the drug into the cells via a receptor-mediated strategy, as the receptors on macrophages are overexpressed during infections. However, the degree of receptor overexpression in infected AMs is not yet fully understood. The design of receptor-specific antibiotic delivery into AMs will provide potential options for future clinical applications if challenges are overcome and appropriate standard processes are established.

The interactions of decorated nanoparticles with the mucus barrier and mucociliary clearance mechanisms in the lungs of patients with COPD and CF have not yet been fully elucidated. Consequently, further investigations are warranted to overcome these limitations and evaluate their clinical applicability.

Following inhalation, nanoparticles rapidly interact with pulmonary surfactant proteins, phospholipids, and other components of the respiratory tract lining fluid, resulting in the formation of a biomolecular corona (Hou et al. 2021; Pervizaj-Oruqaj et al. 2024). This corona can modify the surface characteristics of the decorated nanoparticles, mask targeting ligands, and impair receptor-mediated recognition and cellular uptake, ultimately reducing targeting efficiency.

Furthermore, AMs heterogeneity (Aegerter et al. 2022) represents an important consideration for macrophage-targeted therapies, as tissue-resident alveolar macrophages and recruited monocyte-derived alveolar macrophages exhibit distinct receptor expression profiles, activation states, and phagocytic capacities, particularly during chronic infection and inflammation, which may significantly influence nanoparticle uptake and therapeutic efficacy. Moreover, the pharmacokinetics of antibiotics within AMs are complex, and achieving intracellular drug concentrations that exceed the MIC for prolonged periods remains a significant obstacle to effective treatment of intracellular pathogens. Therefore, careful design and optimization of surface-functionalized nanoparticles are essential to overcome these challenges and preserve their targeting capability.

### 7.2. Strategies to overcome the ongoing challenges

*i). Design of antibiotic carriers with ligands to target specific receptors*: Antibiotic molecules/carriers need to be designed with ligands in such a way that they can selectively bind with the AMs receptors and release the drug into the infected cells. Therefore, modification of the carrier surface is essential to target specific cells, and the interaction between the ligand and receptor enhances the uptake of the delivered drug into infected cells. Therefore, by designing inhalable antibacterials with appropriate ligands targeting specific receptors (Table 3), efficient killing of intracellular-resistant bacteria can be achieved. This information will aid in the synthesis of appropriate antibiotic carriers with ligands that target specific AM receptors.

**Table 3.** AMs receptors and some ligands for targeting AMs (Chae et al. 2023; Branco et al. 2024).

Receptors	Ligands
Lectin (mannose, galactose, sialic acid) receptors	Carbohydrate, glycoprotein, *N*-acetylglucosamine 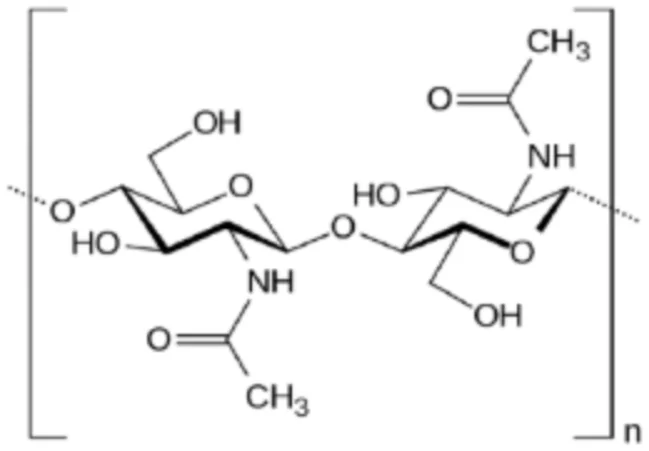
Glucan receptor	*β*-(1→3) Glucans 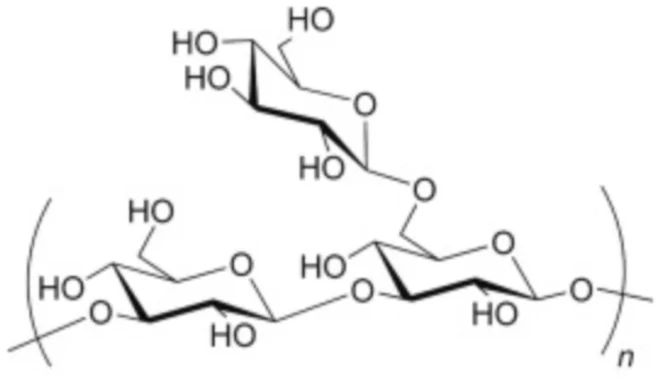
Hyaluronic acid receptor	Hyaluronan-binding proteins (hyaladherins)
Peptidoglycan	Tannic acid 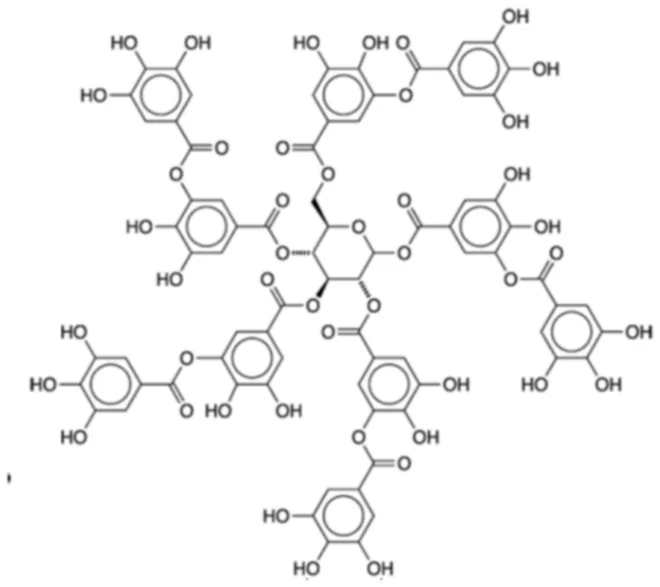 Boronic acid derivatives 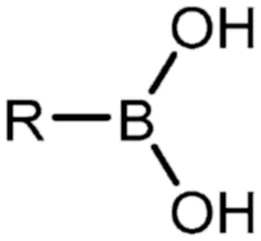
Lipopolysaccharide	Peptides, proteins, antibody
Somatostatin receptors (SSTRs)	Somatostatin neuropeptide (SST)
Folate receptor	Folic acid 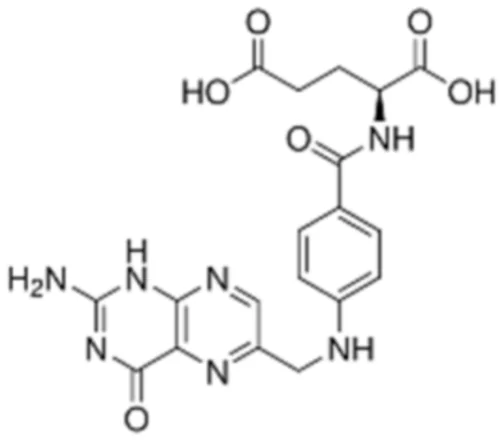

A flowchart for designing antibacterials, pulmonary delivery of the developed formulations, antibiotic deposition into deep lungs, and subsequent killing of intracellular bacteria is presented in Figure 5. However, physicochemical properties, stability, in vitro/in vivo cell culture experiments, and cytotoxicity studies must be performed to ensure safety and efficacy in future preclinical and clinical studies. Following the successful completion of these investigations, comprehensive assessment of translational factors, including large-scale manufacturing feasibility, inhalation device compatibility, and regulatory compliance, will be essential to support the clinical development and commercialization of these targeted inhaled antibiotic delivery systems.

**Figure 5.**
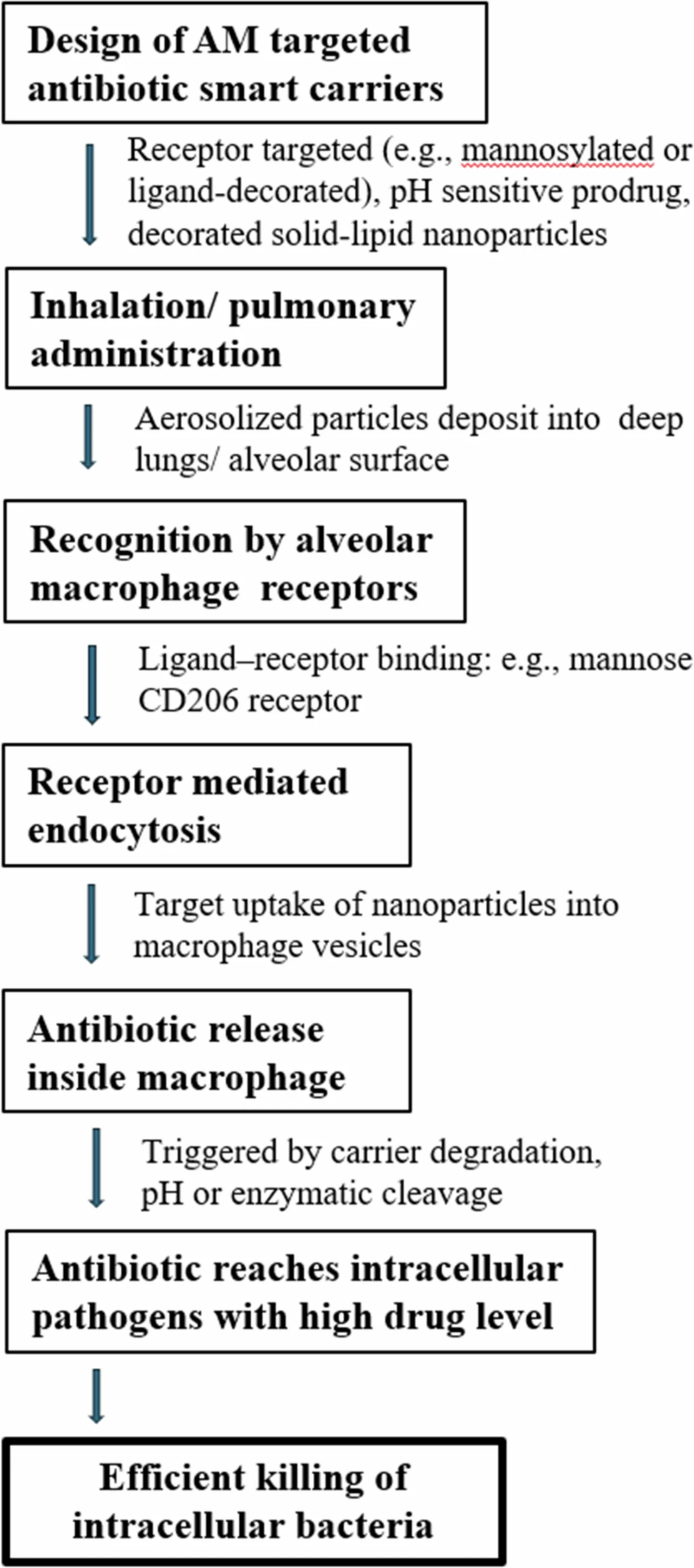
A flow chart of designing AM targeted inhaled antibacterials for efficient killing of intracellular resistant bacteria. The development of macrophage-targeted inhaled antibiotics should begin with the rational design of particles capable of recognizing specific receptors expressed on alveolar macrophages while maintaining suitable aerosolization properties for deep lung deposition. Following inhalation, the particles bind to the target receptors and undergo cellular internalization. Subsequent intracellular antibiotic release within macrophages enables efficient delivery of the drug to the site of infection, thereby enhancing the killing of intracellular bacteria and improving therapeutic efficacy.

*ii). Combination therapy:* Combination antibacterial therapy for lower respiratory tract infections is considered to provide broad-spectrum coverage, improve treatment efficacy against polymicrobial or resistant pathogens, and reduce the risk of treatment failure in severe or complicated cases. As LRTIs are often associated with multiple bacterial pathogens, particularly *Mycobacterium* spp. and *Burkholderia* spp., effective treatment frequently requires combination antibiotic therapy. Incorporating multiple antibiotics within a single delivery system may offer an attractive therapeutic approach to improve therapeutic outcomes (Rakhshan et al. 2023). Furthermore, co-delivery of antibiotics with adjunctive agents, such as mucolytics, biofilm-disrupting compounds, or other adjuvant molecules, can enhance drug penetration into biofilms, reduce the impact of hypersecreted mucus, and improve antibacterial efficacy against persistent and resistant bacteria (Islam and Collet 2026). Although such multifunctional formulations have not yet reached clinical practice, substantial research efforts are underway (Hong et al. 2024; Suraweera et al. 2024) to develop and evaluate these advanced therapeutic systems.

Biofilm-associated respiratory infections are characterized not only by bacterial persistence but also by chronic immune dysregulation, in which prolonged M1 macrophage activation can contribute to excessive inflammation and tissue damage (Labonte et al. 2014). To overcome this challenge, the macrophage-targeted nanoparticle systems should be designed to balance antimicrobial efficacy with immunomodulation. Potential strategies include a) co-delivery of anti-inflammatory or pro-resolving agents to limit excessive M1-driven inflammation while preserving host defense, and b) surface modifications that promote a controlled transition from pro-inflammatory (M1) to reparative (M2) macrophage phenotypes following bacterial clearance (Andrade et al. 2021). The optimal nanoparticle design may differ between acute infections and chronic biofilm-associated diseases, where sustained inflammatory responses are a major contributor to pathology (Andrade et al. 2021; Huang et al. 2024). Thus, future translational studies should evaluate both antimicrobial and immunomodulatory outcomes to ensure effective bacterial eradication without exacerbating chronic airway inflammation.

*iii). Ensure safety of the macrophage-targeted antibiotics*: Toxicity and safety issues are major concerns associated with newly developed drug formulations. In vitro cell culture studies carried out for preliminary investigations are often impractical for answering mechanistic questions. AMs play a critical homeostatic role in the lungs, and AM-targeted antibiotics may modulate intrinsic function, which is unknown. There is still much to learn about the AM microenvironment, receptors of infected cells, and how these cells are regulated, and their functions can be modulated by AM-targeted antibiotic delivery technology. Exclusive studies of macrophage-based formulations in animal models (mice, rats, and rabbits) of bacterial infections, selection of appropriate animals, and infected macrophages for antibiotic delivery are paramount to resolve these questions. Ensuring the safety of AM-targeted inhaled antibiotics requires a combination of rational drug design, careful delivery engineering, and rigorous pre-clinical testing. The goal is to deliver high concentrations to macrophages while avoiding lung injury, systemic toxicity, or unintended immune activation.

Overall, the clinical translation of inhaled antibacterial systems targeting AMs will require overcoming several important biological, technological, and regulatory challenges. Key knowledge gaps include a limited understanding of nanoparticle interactions with the diseased lung microenvironment, AM heterogeneity during infection and inflammation, and the intracellular fate of both nanoparticles and encapsulated antibiotics after macrophage uptake. Furthermore, achieving efficient mucus penetration, sustained intracellular antibiotic concentrations above the minimum inhibitory concentration, scalable manufacturing, and long-term safety remains challenging. Future efforts should therefore focus on the rational design of clinically translatable formulations, the establishment of predictive preclinical models that better reflect human respiratory diseases, and comprehensive evaluation of pharmacokinetics, efficacy, and safety to facilitate successful clinical implementation of AM-targeted inhaled antibacterial therapies.

## 8. Conclusion

LRTIs caused by resistant bacteria, which reside in macrophages, are extremely difficult to treat using conventional antibiotics. The currently available treatment systems or antibiotic dosage forms are incapable of producing efficient outcomes because of the limited availability of the delivered antibiotics into the AMs. High doses of antibiotics in oral or injectable dosage forms cause severe off-target dose-related adverse effects. Drug delivery technologies targeting macrophages are emerging. As inhaled drug delivery technology efficiently delivers drugs into locally infected lung cells, antibiotic delivery following targeting AMs would produce a new window for selectively killing intracellular bacteria and reducing morbidity and mortality. Future studies should focus on engineering mucus-penetrating and retention-optimized decorated nanoparticles while systematically investigating their interactions with the altered mucus barriers and mucociliary clearance mechanisms in COPD and CF lungs, thereby enhancing pulmonary drug delivery efficiency and facilitating their clinical translation. The formation of a biomolecular corona on inhaled nanoparticles can reduce AM targeting efficiency; therefore, rational particle design is essential to minimize corona formation and preserve targeting functionality. Alveolar macrophage heterogeneity poses a challenge for macrophage-targeted therapies, as tissue-resident and monocyte-derived AMs exhibit distinct receptor expression patterns, activation states, and phagocytic capacities that can influence nanoparticle uptake and therapeutic efficacy. Furthermore, the complex intracellular pharmacokinetics of antibiotics within AMs hinder the maintenance of drug concentrations above the minimum inhibitory concentration (MIC) for prolonged periods; therefore, future research should focus on the rational design and optimization of surface-functionalized nanoparticles to enhance targeting efficiency and intracellular drug delivery. There is much to learn regarding inhaled AM-targeted antibiotic delivery strategies. Exclusive preclinical and clinical studies are warranted to ensure the safety and stability of AM-targeted inhaled antibiotic formulations for the complete eradication of intracellular resistant bacteria. As a compelling therapeutic strategy, AM-targeted inhaled antibiotic delivery technology might be one way to overcome the ongoing challenges and ensure efficient treatment of not only lung infections but also other diseases.

## Data Availability

Data sharing is not applicable to this article as no new data were created or analyzed in this study.
